# Supplementary material for: Divergent Resistance Mechanisms to Immunotherapy Explain Responses in Different Skin Cancers
Source: Cancers (Basel). 2020 Oct 13;12(10):2946. doi: 10.3390/cancers12102946 (PMC7599806; doi:10.3390/cancers12102946)
Supplement: Supplementary file 1 [file cancers-12-02946-s001.pdf]

Supplementary Materials: Divergent Resistance Mechanisms to Immunotherapy Explain Responses in Different Skin Cancers

Emmanuel Dollinger, Daniel Bergman, Peijie Zhou, Scott X. Atwood and Qing Nie

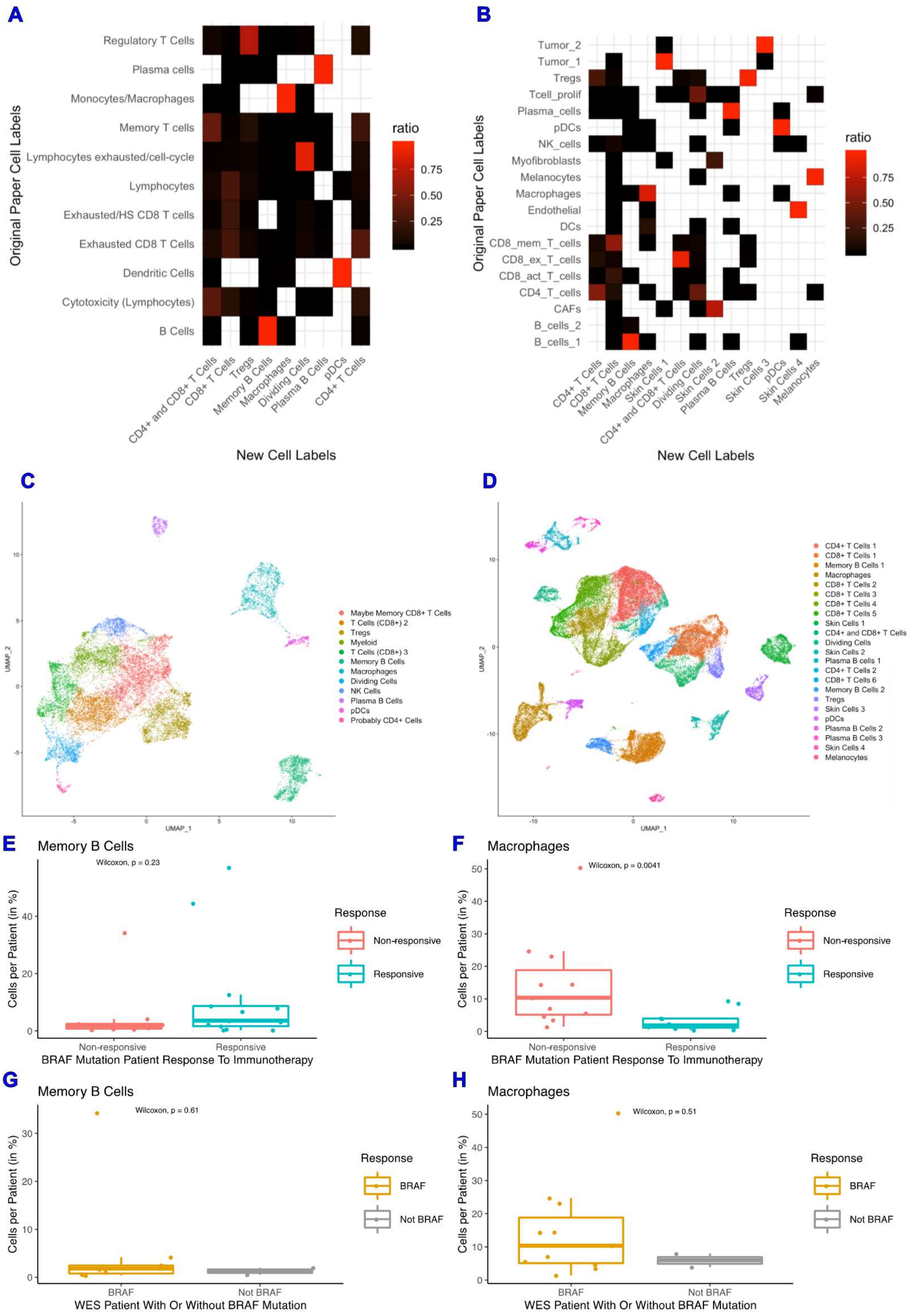

Figure S1. Comparison of cluster identification to original cluster labels and BRAF comparisons. (**A,B**): Heatmaps representing the fraction of our clusters in each cluster in the original paper for

melanoma (**A**) and BCC (**B**). Each column adds to 1. If an original cluster did not contain cells from a new cluster, that space is left blank. (**C,D**): Dimensionality reduction of the melanoma dataset (**C**) and the BCC dataset (**D**), with their original cluster labels. Compare with Fig 1 A and B respectively. (**E,F**) Comparison of percent of memory B cells and macrophages in responders and non-responders with a BRAF mutation. (**G,H**) Comparison of percent of memory B cells and macrophages in patients with and without a BRAF mutation.

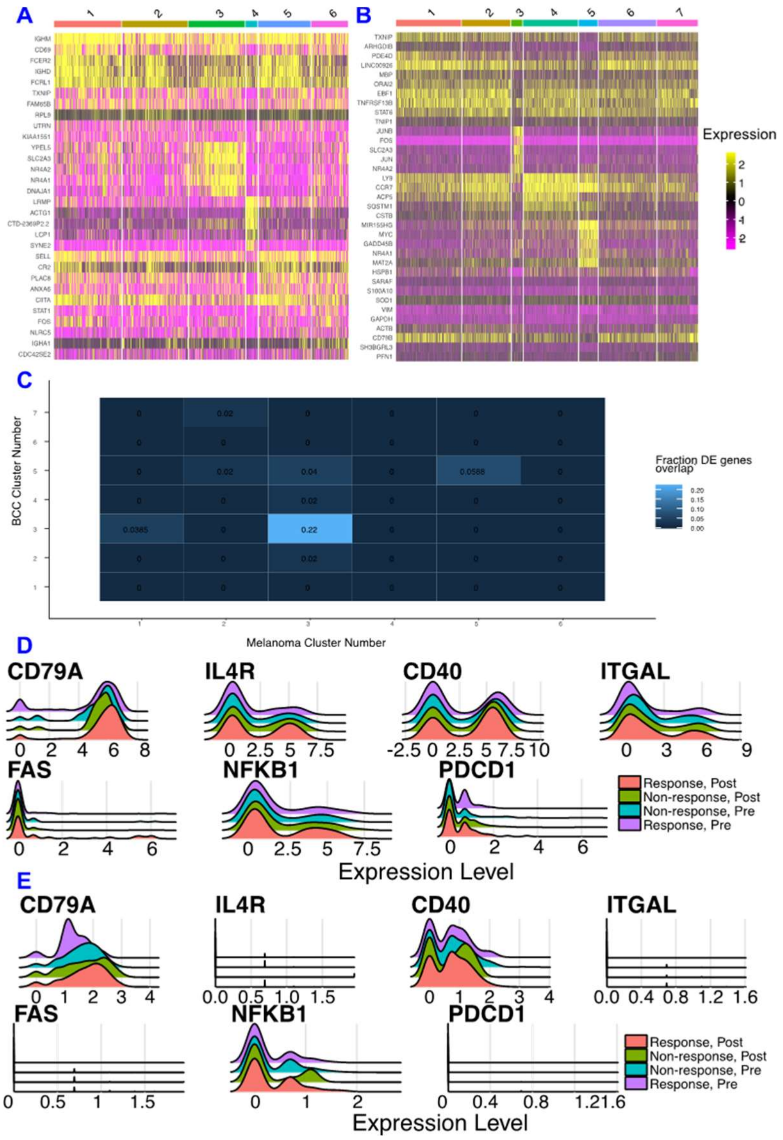

Figure S2. Comparison of memory B cells subsets. (**A,B**) Differential expression of memory B cells in melanoma (**A**) and BCC (**B**). (**C**) Fraction of differentially expressed genes in a melanoma and BCC cluster. (**D,E**) Expression of markers used to calculate the activation and exhaustion score of melanoma (**D**) and BCC (**E**).

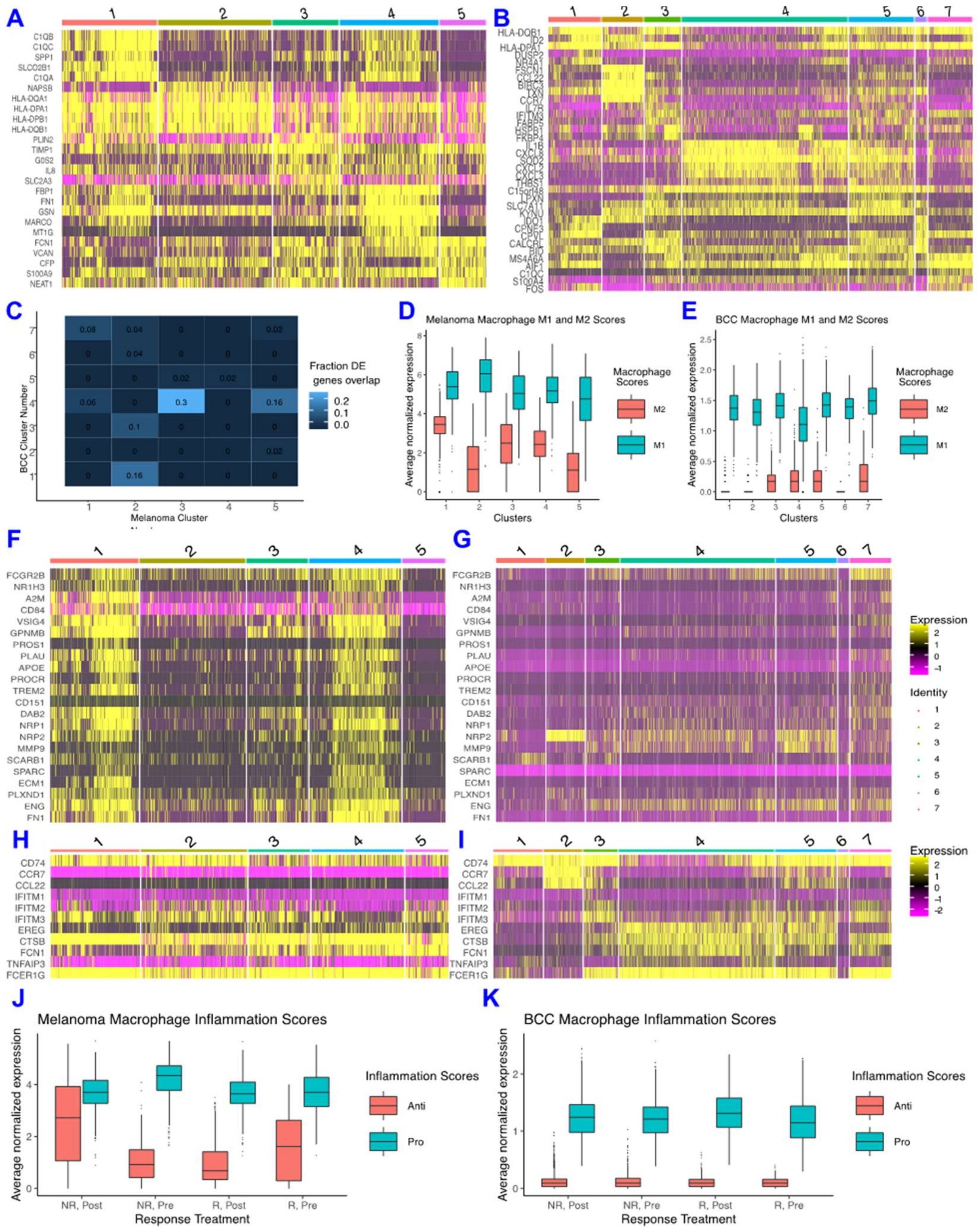

Figure S3. Comparison of macrophage subsets. (**A**,**B**) Differential expression of the melanoma macrophage clusters (**A**) and the BCC macrophage clusters (**B**). (**C**) Fraction of differentially expressed genes in a melanoma and BCC cluster. (**F**,**G**) Expression of anti-inflammatory genes in melanoma macrophages (**F**) and BCC macrophages (**G**). (**H**,**I**) Expression of pro-inflammatory genes in melanoma macrophages (**H**) and BCC macrophages (**I**). (**J**,**K**) Inflammation scores for melanoma (**J**) and BCC (**K**) macrophages, grouped by response and treatment.

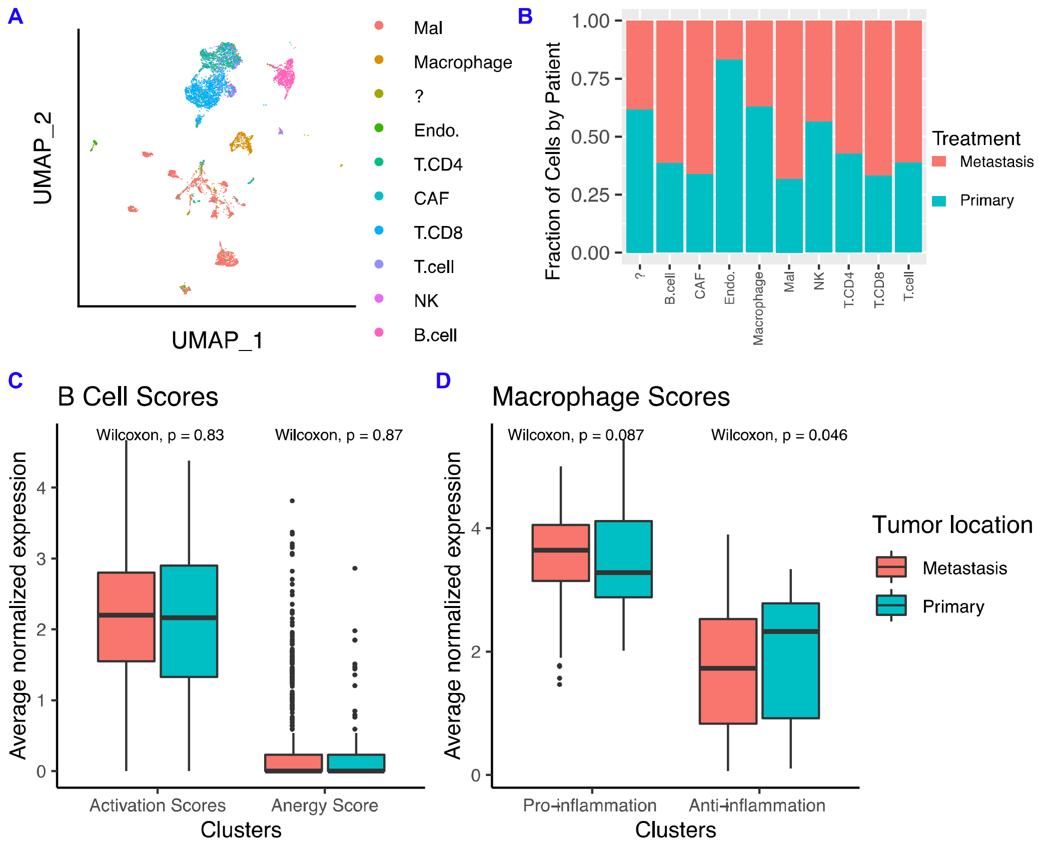

Figure S4. Comparison of immune system at primary and metastatic site of melanoma patients. (**A**) Dimensionality reduction of all cells, using the original paper's cluster labels. (**B**) Distribution of all cell types in the primary and the metastatic sites. (**C**,**D**) B cell activation and anergy scores (**C**) and macrophage pro- and anti-inflammation scores (**D**) by tumor location.

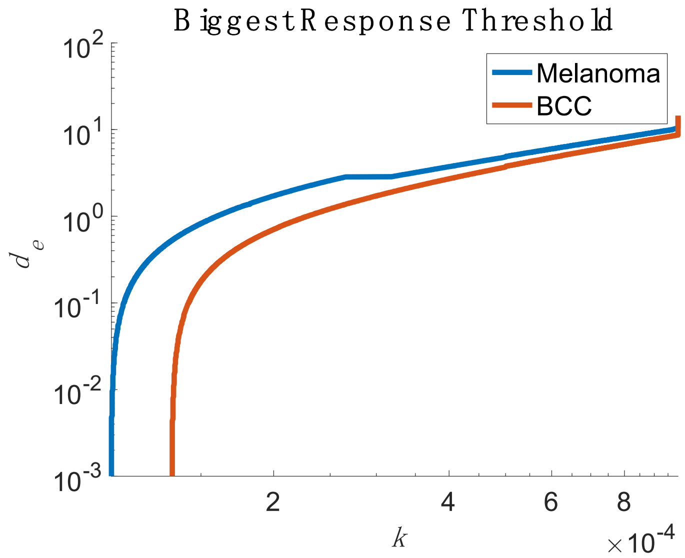

Figure S5: Killing rate at which largest response is observed. For a fixed d_e value, we determine the k value at which the biggest response occurs. We see that melanoma experiences this maximal response at lower values of k indicating that therapy is more likely to result in a response. By varying d_e along the y-axis, we see that this result is robust. To determine the size of the response we sought to maximize, we computed the rate of change of the largest, stable cancer burden as k varied, normalizing for the size of the cancer.

Table S1. Ratios of memory B cells to macrophages in each cancer and response to treatment. The melanoma ratios match the predictions from the model, whereas the BCC ratio pre-treatment does not.

Cancer	Response Treatment	Memory B cells (Number of Cells)	Macrophages (Number of Cells)	Ratio (B/M)
Melanoma	Responder, Pre-treatment	464	160	2.9
Melanoma	Responder, Post-treatment	908	253	3.6
Melanoma	Non-responder, Pre-treatment	50	276	0.18
Melanoma	Non-responder, Post-treatment	50	722	0.07
BCC	Responder, Pre-treatment	13	128	0.10
BCC	Responder, Post-treatment	5547	568	9.8
BCC	Non-responder, Pre-treatment	97	1203	0.08
BCC	Non-responder, Post-treatment	50	722	0.21

Table S2. ODE model parameters.

Name	Description	Value		Units	Source
		Melanoma	BCC		
a	Maximum proliferation rate of tumor cells	0.514		days^{-1}	[1]
b	Inverse carrying capacity of tumor	1.02×10^{-9}		cells^{-1}	[1]
b_e	Maximum memory B cell proliferation rate	1.5		days^{-1}	[2,3]
d	Death rate of memory B cells	2		days^{-1}	[2]
d_e	Maximum rate of cancer-mediated deactivation of memory B cells	1		days^{-1}	Varied as bifurcation parameter
d_m	Death rate of macrophages	3		days^{-1}	Estimated from [4]
g	Source rate of macrophages	30		$\text{cells}\cdot\text{days}^{-1}$	Estimated from [4]
k	Memory B cell killing rate	10^{-4}		$\text{cells}^{-1} \cdot \text{days}^{-1}$	[1,5-7]; Varied as bifurcation parameter
κ_a	EC50 for apoptotic-signaling-induced proliferation of macrophages	4.2×10^7	8.4×10^7	$\text{cells}\cdot\text{days}^{-1}$	[8]; Figure 1
κ_d	EC50 for cancer-mediated upregulation of memory B cell deactivation	2.5×10^6	2.5×10^4	cells	Figure 1
κ_e	EC50 for cancer-mediated upregulation of memory B cell proliferation	500		cells	Estimated; [9-11]
κ_m	EC50 for macrophage-mediated downregulation of memory B cell proliferation	500	11	cells	Estimated
p	Maximum rate of apoptotic-signaling-induced proliferation of macrophages	4		days^{-1}	Estimated
s	Source rate of memory B cells	5×10^3		$\text{cells}\cdot\text{days}^{-1}$	[12,13]

Table 3. Defining relations of non-dimensionalized ODE system. *t* represents the original time variable and τ represents the new time variable.

$x = bC$	$y = \frac{k}{a}B$	$z = \frac{1}{\kappa_m}M$
$\tau = at$	$\alpha = \frac{ks}{a^2}$	$\beta = \frac{d}{a}$
$\gamma = \frac{b_e}{a}$	$\delta = b\kappa_e$	$\epsilon = \frac{d_e}{a}$
$\zeta = b\kappa_d$	$\eta = \frac{g}{a\kappa_m}$	$\theta = p$
$\lambda = b\kappa_a$	$\mu = a$	$\nu = \frac{d_m}{a}$

Table S4. Noise parameters and relationships of state variables to proportionality constants.

State Variable	Additive Proportionality Constant	Multiplicative Proportionality Constant
C	$400a$	$0.4a$
B	$0.04s$	$0.2b_e$
M	$0.04g$	$0.2p$

Model reduction of the four-state model incorporating pro-inflammatory macrophages

Denote the population of pro-inflammatory macrophages as M_1 then we can derive a four-state model with

$$C' = aC(1 - bC) - kCB \tag{1}$$

$$B' = s - dB + b_e \frac{C}{\kappa_e + C} \frac{\kappa_m}{\kappa_m + M} B - d_e \frac{C}{\kappa_d + C} B + b_{e1} \frac{\kappa_{m1} M_1}{\kappa_{m1} + M_1} B \tag{2}$$

$$M' = g - d_m M + p \frac{kCB}{\kappa_a + kCB} M - k_+ M + k_- M_1 \tag{3}$$

$$M_1' = g_1 - d_{m1} M_1 + k_+ M - k_- M_1 \tag{4}$$

Here the additional terms account for the upregulation of anti-tumor immune cells and plastic conversion with anti-inflammatory macrophages by newly added pro-inflammatory macrophages.

We assume that the conversion within macrophages subpopulations is fast in timescale, hence we have the dynamical equilibrium $M_1 = \frac{k_+ M}{k_-}$. Summing up the last two equations and utilizing this relationship, we then have the effective dynamics for anti-inflammatory macrophages

$$M' = K(g + g1) - K(d_m + \frac{k_+ d_{m1}}{k_-})M + Kp \frac{kCB}{\kappa_a + kCB} M, \qquad K = \frac{k_-}{k_+ + k_-}, \tag{5}$$

which has exactly the same form with the M equations in three-state model except that the parameters are reformatted. In the effective B equations, the influence of additional term $b_{e1} \frac{\kappa_{m1} M_1}{\kappa_{m1} + M_1} B$ can also be equivalently reduced into cancer-regulated term $d_e \frac{C}{\kappa_d + C} B$, since they have the similar Hill-function term, and the dynamics of M_1 is positively regulated by C.

Therefore, the three-state model in main text can be viewed as the model reduction as four-state model, where the effect of pro-inflammatory macrophages has been accounted by the existing parameters.

Non-dimensionalization of the dynamical model and parameter selection

We non-dimensionalized our system to simplify the analysis but present our results in terms of these original equations. These are the non-dimensionalized equations (see Supplementary Table 3 for relationships of new variables to old variables):

$$\frac{dx}{d\tau} = x(1 - x) - xy \tag{6}$$

$$\frac{dy}{d\tau} = \alpha - \beta y + \gamma \frac{x}{\delta + x} \frac{1}{1+z} y - \epsilon \frac{x}{\zeta + x} y \tag{7}$$

$$\frac{dz}{d\tau} = \eta - \nu z + \theta \frac{xy}{\lambda + \mu xy} z \tag{8}$$

Equilibria and their stability of deterministic model

In solving for equilibria, we are able to simplify the set of equations into a union of two solution sets:

$$C = 0 \quad \text{or} \quad f(C) = 0 \tag{9}$$

where f is a degree 5 polynomial. Of the six roots, we select only the sensical ones, i.e. those where (C, B, M) lies in the first octant of $\mathbb{R}^3$. We determine the stability of these fixed points using the eigenvalues of the Jacobian, looking for those with all eigenvalues having negative real part. In Figure 4B, we show the number of stable equilibria in the k-d_e plane. We choose to classify the stable fixed points based on the size of the cancer population. When $C = 0$ is stable, this is complete elimination. When C is nonzero but several orders of magnitude smaller than its carrying capacity, we describe this as a dormant state. When C is at least 1% of the carrying capacity, we call this a high cancer state. When two such states are stable, we call the one with the larger cancer population very high.

Adding stochastic effect to the model

We next turned to the reality of biological noise and considered how this could impact our model. Let X_t be the state vector of our system at time t and let $b(X_t)$ be our time-independent ODE function. We take a generic, time-independent noise term, $\sigma(X_t)$ and generated the following SDE model:

$$dX_t = b(X_t)dt + \sigma(X_t)dW_t \tag{10}$$

with W_t being a standard Weiner process. For our noise term, σ, we assume there are both additive and multiplicative sources of noise for each population. These are given by independent Weiner processes and so σ is a 3×6 matrix. For determining the parameters of these functions, we chose them to be proportional to the parameters of our ODE system (see table below). For B cells and macrophages, these choices were tied to their source rates (additive coefficient) and proliferation rates (multiplicative coefficient). The energy landscapes shown in Figure 5A scaled these noise terms by $1/a$. The matrix σ is (see Supplementary Table 4 for relationships of state variables and additive and multiplicative proportionality constants):

$$\sigma(C, B, M) = \begin{bmatrix} 400a & 0 & 0 & 0.4a \cdot C & 0 & 0 \\ 0 & 0.04s & 0 & 0 & 0.2b_e \cdot B & 0 \\ 0 & 0 & 0.04g & 0 & 0 & 0.2p \cdot M \end{bmatrix} \tag{11}$$

References:

1. Pillis, L.G. de; Radunskaya, A.E.; Wiseman, C.L.A. Validated Mathematical Model of Cell-Mediated Immune Response to Tumor Growth. *Cancer Res.* **2005**, *65*, 7950–7958.
2. Conway, J. M. and Perelson, A. S. A hepatitis C virus infection model with time-varying drug effectiveness: solution and analysis. *PLoS Comput. Biol.* **2014**, *10*, e1003769.
3. Davenport, M. P.; Ribeiro, R. M.; Chao, D. L. and Perelson, A. S. Predicting the impact of a nonsterilizing vaccine against human immunodeficiency virus. *J. Virol.* **2004**, *78*, 11340–11351.
4. Murdoch C.; Muthana M.; Coffelt S.B. Mathematical modeling and stability analysis of macrophage activation in left ventricular remodeling post-myocardial infarction. *BMC Genomics* **2012**, *13*, S21.

5. Elemans, M.; Florins, A.; Willems, L. and Asquith, B. Rates of CTL killing in persistent viral infection in vivo. *PLoS Comput. Biol.* **2014**, *10*, e1003534.
6. Ganusov, V.V.; Goonetilleke*et*, N.; Liu, M.K.P.; Ferrari, G.; Shaw, G.M.; McMichael, A.J.; Borrow, P.; Korber, B.T.; Perelson, A.S. Fitness costs and diversity of the cytotoxic T lymphocyte (CTL) response determine the rate of CTL escape during acute and chronic phases of HIV infection. *J. Virol.* **2011**, *85*, 10518–10528.
7. Wick, W. D.; Yang, O. O.; Corey, L.; Self, S. G. How many human immunodeficiency virus type 1-infected target cells can a cytotoxic T-lymphocyte kill? *J. Virol.* **2005**, *79*, 13579–13586
8. Murdoch, C.; Muthana, M.; Coffelt, S. B.; Lewis, C. E. The role of myeloid cells in the promotion of tumour angiogenesis. *Nature Reviews Cancer* **2008**, *8*, 618–631, doi:10.1038/nrc2444.
9. Cabrita, R.; Lauss, M.; Sanna, A.; Donia, M.; Larsen, M.S.; Mitra, S.; Johansson, I.; Phung, B.; Harbst, K.; Vallon-Christersson, J.; et al. Tertiary lymphoid structures improve immunotherapy and survival in melanoma. *Nature* **2020**, *577*, 1–5.
10. Petitprez, F.; Reyniès,A.D.; Keung,E.Z.; Chen, T.W.; Sun, C.M.; Calderaro, J.;Jeng, Y.M.; Hsiao, L.P.; Lacroix, L.; Bougouin, A.; et al. B cells are associated with survival and immunotherapy response in sarcoma. *Nature* **2020**, *577*, 556–560.
11. Helmink B A, Reddy S M, Gao J, B cells and tertiary lymphoid structures promote immunotherapy response. *Nature* **2020**, *577*, 549–555.
12. Althaus, C. L. and De Boer, R. J. Dynamics of immune escape during HIV/SIV infection. *PLoS Comput. Biol.* **2008**, *4*, e1000103
13. De Boer, R. J. Understanding the failure of CD8+ T-cell vaccination against simian/human immunodeficiency virus. *J. Virol.* **2007**, *81*, 2838–2848
